# The map of bone metastasis in nasopharyngeal carcinoma: A real‐world study

**DOI:** 10.1002/cam4.6383

**Published:** 2023-08-10

**Authors:** Dong‐Hua Luo, Jia‐Xin Li, Wan‐Ping Guo, Chen‐Guang Guo, Xiao‐Han Meng, Pei‐Jun Xie, Jie‐Yi Lin, Hao‐Yuan Mo, Qun Zhang, Yong Chen, Guo‐Ping Shen

**Affiliations:** ^1^ Department of Nasopharyngeal Carcinoma Sun Yat‐sen University Cancer Center Guangzhou Guangdong PR China; ^2^ Department of Radiation Oncology The First Affiliated Hospital of Sun Yat‐sen University Guangzhou Guangdong PR China; ^3^ Department of Oncology The First Affiliated Hospital of Guangdong Pharmaceutical University, Guangdong Pharmaceutical University Guangzhou Guangdong PR China

**Keywords:** bone metastasis, distant metastasis, nasopharyngeal carcinoma

## Abstract

**Objectives:**

The aim of this study was to compare the metastatic patterns of synchronous bone metastasis (SBM) and metachronous bone metastasis (MBM) in nasopharyngeal carcinoma (NPC).

**Methods:**

This study included bone metastases in NPC patients from 2005 to 2016 in a Chinese hospital. Cohort 1 was collected from 2005 to 2010 for discovery, and Cohort 2 from 2011 to 2016 for validation. The chi‐squared test, Wilcoxon rank sum test, and Kaplan–Meier technique were used to compare site, time, and survival between cohorts 1 and 2. Prognostic factors were analyzed using univariate or multivariate Cox regression.

**Results:**

Cohort 1 had 112 individuals with SBM and 394 with MBM, and cohort 2 had 328 with SBM and 307 with MBM. The thoracic vertebra was the most frequently affected site of metastasis. Patients with SBM more often had metastasis to the cervical vertebrae compared with patients with MBM (34.5% vs. 22.3%, *p* < 0.05). Patients with SBM had better overall survival (42.2 months, 95% CI: 33.9–50.7) than patients with MBM (24.9 months, 95% CI: 22.2–28.7). Age at bone metastasis detection, metastasis to other organs, and more bone metastasis locations were associated with worse prognosis. The majority of MBMs occurred at 7 to 18 months after NPC diagnosis.

**Conclusion:**

Radiotherapy does not modify the metastatic patterns of NPC bone metastases. Patients with SBM tend to have metastasis to the cervical vertebra, which is close to the nasopharynx. Paying more attention to bone metastases during follow‐up in the first 2 years after an NPC diagnosis.

## 1 INTRODUCTION

Nasopharyngeal carcinoma (NPC) is an epithelial carcinoma arising from the nasopharyngeal mucosal lining. With advances in treatment strategies over the course of the last 20 years, the cure rate of NPC has increased significantly. The application of magnetic resonance imaging (MRI), intensity‐modulated radiotherapy, and neoadjuvant chemotherapy has improved the overall survival (OS), progression‐free survival (PFS), and locoregional relapse‐free survival (LRFS) of NPC, whereas distant metastasis‐free survival showed little improvement. The main pattern of treatment failure in NPC has shifted from recurrence to distant metastasis.^1^

Distant metastasis of NPC includes both synchronous metastasis (smNPC), that is, metastasis at the time of initial diagnosis, and metachronous metastasis (mmNPC), that is, metastasis after radical radiotherapy. The wider application of [18F] fluorodeoxyglucose positron emission tomography and computed tomography (PET/CT) has revealed that up to 14.8% of patients with NPC have synchronous metastasis.^2^ In addition, 15%–22% of patients diagnosed with NPC without any clinical evidence of distant metastasis will display metachronous metastasis, usually within 5 years.^3^, ^4^ Except for these two metastases that occur in different disease stages before or after treatment, most researchers have focused on building prognostic models to better predict survival risk in patients with smNPC.^5^, ^6^ Few studies have compared patterns of metastases in NPC patients with synchronous compared with metachronous metastases.

NPC metastasis to distant organs is common in the bones, lungs, liver, and distant lymph nodes. Metastatic NPC with bone involvement is common, accounting for more than 50% of metastatic cases.^7^, ^8^ The location and number of metastatic lesions both have significant effects on survival.^9^, ^10^, ^11^ A retrospective study examined patients with NPC and bone‐only metastases, who were stratified according to the location and number of specific bone metastatic sites, in order to identify patients who may benefit from combined chemoradiotherapy.^12^ To date, no study has specifically evaluated the difference between synchronous bone metastasis (SBM) at the time of initial diagnosis and metachronous bone metastasis (MBM) after radical radiotherapy.

Therefore, we conducted a large cohort study to compare the metastatic patterns of synchronous and metachronous bone metastasis in an attempt to better understand the characteristics of smNPC and mmNPC, which may provide evidence for the individualized treatment of bone metastatic NPC.

## 2 MATERIALS AND METHODS

### 2.1 Patient selection

Patients newly diagnosed with NPC between 2005 and 2016 at the Sun Yat‐Sen University Cancer Center were analyzed. These patients were obtained from a prospective observational cohort that was registered in the Chinese Clinical Trial Registry (ChiCTR, http://www.chictr.org.cn/, ChiCTR‐ROC‐17012658) and approved by the Human Ethics Approval Committee of Sun Yat‐sen University Cancer Center (No. YB2005001). We recruited patients with pathologically confirmed NPC for whom complete medical information were available. The patients entered our analysis if those suffer bone metastasis regardless of whether those have other organs metastasis or not.

### 2.2 Stage

All patients underwent a physical examination, hematological and biochemical analysis, fiberoptic nasopharyngoscopy, and MRI of the nasopharynx and neck (or a CT scan if MRI is contraindicated) as per the pretreatment evaluation. The systemic staging workup included chest X‐ray or thoracic CT scan, abdominal ultrasonography or abdominal CT scan, and emission computed tomography (ECT) of the bone. A PET/CT scan would be performed in exceptional cases if a patient preferred the self‐financed investigation to conventional staging investigations. The patients were restaged according to the 7th American Joint Committee on Cancer edition of the TNM Cancer Staging system.^13^

### 2.3 Definition of bone metastasis region

The bone metastasis sites were divided into two groups. The first was the midline bone, which includes five regions, that is, the sternum, cervical vertebra, thoracic vertebra, lumbar vertebra, and sacral vertebra. The second was the didymous bone, which includes seven regions, that is, the skull, clavicle, scapula, humerus, rib, pelvic bone, and femur. The first discovery was the recording of bone metastasis regions at the initial imaging examination with positive events. The subsequent discovery was defined as recording new bone metastasis region occuring after first discovery during the follow‐up period.

### 2.4 Treatment

Patients with stage M0 disease underwent definitive radiotherapy. The dose ranges for the nasopharynx, node‐positive region, and node‐negative region were 68–72 Gy, 60–68 Gy, and 50–60 Gy, respectively. The radiation doses were delivered approximately 2 Gy per fraction, from Monday to Friday, five times per week. The radiotherapy methods included two‐dimensional conventional radiotherapy (2D‐CRT) and intensity‐modulated radiotherapy (IMRT). Patients with stage I or II tumors received radiotherapy alone. Patients with stage III–IV cancer underwent radiotherapy and chemotherapy with cisplatin‐based regimens.

The common cisplatin‐based chemotherapy regimens were as follows: PF regimen: cisplatin (20‐30/m^2^ on d1‐3) combined with 5‐fluorouracil (4–5 g/m^2^, 120 h continuous intravenous infusion); TP regimen: docetaxel (70–80 mg/m^2^ d1) combined with cisplatin (20–25 mg/m^2^ d1–3); TPF regimen: docetaxel (60–70 mg/m^2^ d1) combined with cisplatin (20–25 mg/m^2^ d1–3) plus 5‐fluorouracil (3–3.75 g/m^2^, 120 h continuous intravenous infusion); and GP regimen: gemcitabine (0.8–1 g/m^2^ d1,8) and cisplatin (20–30 mg/m^2^ d1–3). Concurrent chemotherapy was scheduled on Days 1, 22, and 43 with 80 to 100 mg/m^2^ of cisplatin for 1 or 3 days per cycle during radiotherapy. Chemotherapy was postponed or discontinued for patients who experienced serious adverse events and could not recover before the next scheduled cycle. Chemotherapy was performed based on 3‐week intervals.

The same cisplatin‐based chemotherapy as above is the most used treatment regimen for patients with metastatic NPC. Radiotherapy targeting both the primary tumor and the regional lymph nodes was administered to some patients as part of a multidisciplinary approach. Local treatment of metastatic lesions, including radiotherapy, surgical resection, ablation, and other treatments, has been applied in some patients to control local symptoms and eliminate metastases in the bone, liver, lung, or other organs.

### 2.5 Follow‐up

After treatment, the patients were assessed every 3 months during the first 2 years of follow‐up, every 6 months for at least 5 years, and annually thereafter until death. Follow‐up examinations included nasopharyngoscopy, MRI of the head and neck, and imaging of the chest and abdomen. Bone ECT or PET/CT was performed when signs of recurrence or metastasis were observed. The last follow‐up was in December 2021.

### 2.6 Statistical analysis

Differences in the distribution of bone metastasis between SBM and MBM were estimated using the chi‐squared test. The endpoint was overall survival (OS), which was defined as the interval between the date of diagnosis of bone metastases and the date of the last visit or death. Calculations were also made for the time to metastasis, which was determined as the period of time between the NPC diagnosis date and the date that bone metastases were found (Figure S1). The distribution of time to metastasis was compared by the *t*‐test (normal distribution) or Wilcoxon rank sum test (non‐normal distribution). For variables presenting a non‐normal distribution, the Box‐Cox change could convert them to a normal distribution, which could then be tested for modified variable normality using the skewness test, kurtosis test, and equal variances test, and finally compared by the *t*‐test. Survival outcomes were analyzed using the Kaplan–Meier method. Univariate and multivariate Cox regression analyses were performed to identify significant prognostic factors for survival outcomes and to calculate their corresponding hazard ratios (HRs). Statistical significance was set at *p* < 0.05. Statistical analyses were performed using the R studio software package.

## 3 RESULTS

### 3.1 Patient characteristics

**FIGURE 1:**
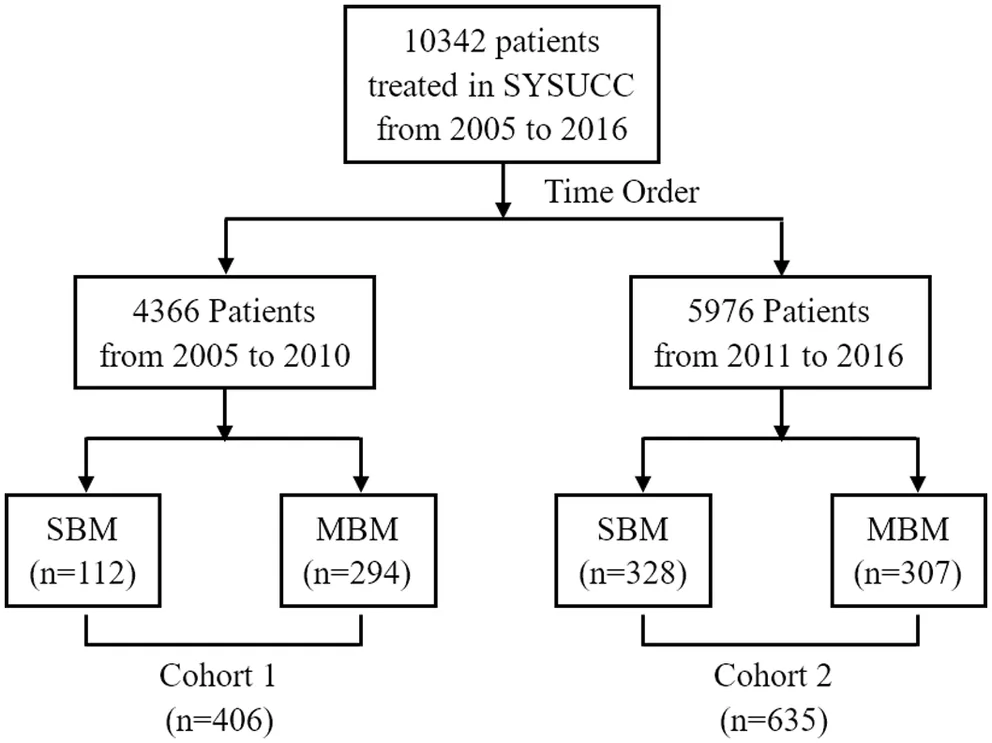
Follow chart of study design. A total of 10,342 patients newly diagnosed with NPC were analyzed. Cohort 1 which was used as a discovery sample included patients enrolled between 2005 and 2010. Cohort 2 which was used as a validation sample included patients enrolled between 2011 and 2016. MBM, metachronous bone metastasis; SBM, synchronous bone metastasis; SYSUCC: Sun Yat‐sen University Cancer Center.

During the study period, 10,342 patients newly diagnosed with NPC were analyzed. Cohort 1 (discovery sample) included patients with SBM (*n* = 112) or MBM (*n* = 394) enrolled between 2005 and 2010. Cohort 2 (validation sample) also included patients with SBM (*n* = 328) or MBM (*n* = 307) enrolled between 2011 and 2016 (Figure 1). The median follow‐up times were 22.0 and 21.7 months in cohorts 1 and 2, respectively. Table S1 summarizes the patient characteristics, including sex, age, clinical stage, treatment regimen, site of metastasis, and follow‐up times, for both cohorts 1 and 2. In both cohorts, metastasis to other organs was significantly more common in patients with SBM compared with patients with MBM. From 2005 to 2011, the methods of radiotherapy have been changed. The proportion of intensity‐modulated radiation therapy (IMRT) increased gradually, those were 6.1% and 71.3% in cohort 1 and cohort 2, respectively (Table S2).

### 3.2 Distribution of bone metastasis sites

**FIGURE 2:**
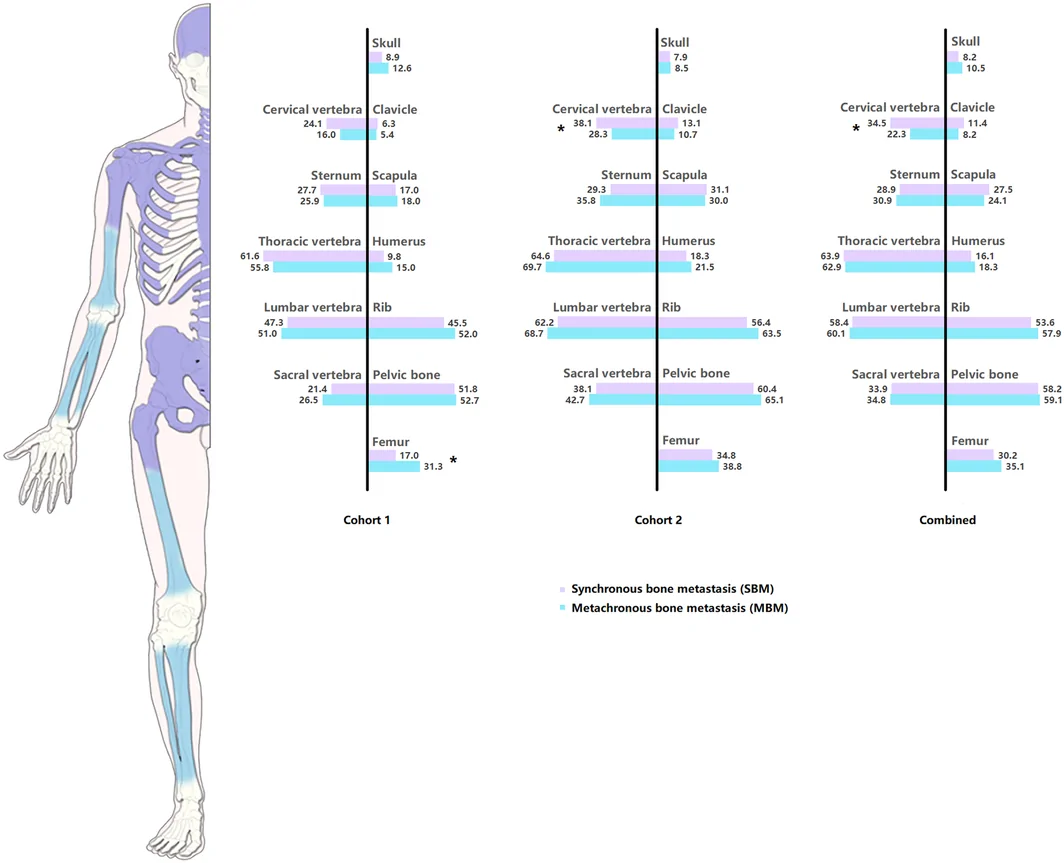
Incidence of bone metastasis in 11 skeleton regions. In midline bone, *p*‐values calculated by the chi‐squared test between SBM and MBM in cervical vertebra, sternum, thoracic vertebra, lumbar vertebra and sacral vertebra are 0.058, 0.709, 0.289, 0.505, and 0.289 in cohort 1, 0.009, 0.078, 0.174, 0.084, and 0.242 in cohort 2, <0.001, 0.469, 0.749, 0.591, and 0.760 in combined cohort, respectively. In didymous bone, *p*‐values calculated by the chi‐squared test between SBM and MBM in skull, clavicle, scapula, humerus, rib, pelvic bone, and femur are 0.303, 0.753, 0.802, 0.176, 0.241, 0.866, and 0.004 in cohort 1, 0.803, 0.360, 0.757, 0.311, 0.068, 0.213, and 0.295 in cohort 2, 0.211, 0.081, 0.218, 0.362, 0.171, 0.774, and 0.098 in combined cohort, respectively. *p < 0.05.

We detected 1444 and 2974 bone metastatic regions, in cohorts 1 (*n* = 406) and 2 (*n* = 635), respectively. In cohort 1, the vertebral column was the most common site of involvement (*n* = 612, 42.4%), followed by pelvic bone (*n* = 213, 14.8%), ribs (*n* = 204, 14.1%), femur (*n* = 111, 7.7%), and sternum (*n* = 107, 7.4%). This pattern was also evident for cohort 2, with involvement of the vertebrae (*n* = 1309, 44.0%), pelvic bone (*n* = 398, 13.4%), ribs (*n* = 380, 12.8%), femur (*n* = 233, 7.8%), and sternum (*n* = 206, 6.9%). For all skeletal metastatic regions, cervical vertebral metastases were significantly more common in the SBM group compared with the MBM group (*p* < 0.001) (Table 1). In both cohorts, the thoracic vertebra was the most common site of bone metastasis, followed by the lumbar vertebra, pelvic bone, rib, sternum, and sacral vertebra (Figure 2). The bone metastasis‐accumulating regions were further studied (Table 2). Follow‐up for new bone metastases in patients with SBM was significantly more common in cohort 1. In a combined analysis of both cohorts, we found no significant differences in the distribution of accumulated regions between the SBM group and the group MBM.

**TABLE 1:** Bone metastasis sites distribution in synchronous or metachronous bone metastasis patients with nasopharyngeal carcinoma.

Bone metastasis site	Cohort 1			Cohort 2			Combined		
	SBM	MBM	*p*‐value^a^	SBM	MBM	*p*‐value^a^	SBM	MBM	*p*‐value^a^
	*N* = 379 (%)	*N* = 1065 (%)		*N* = 1490 (%)	*N* = 1484 (%)		*N* = 1869 (%)	*N* = 2549 (%)	
Midline bone									
Cervical vertebra	27 (7.1)	47 (4.4)	0.040	125 (8.4)	87 (5.9)	0.007	152 (8.1)	134 (5.3)	<0.001
Sternum	31 (8.2)	76 (7.1)	0.505	96 (6.4)	110 (7.4)	0.298	127 (6.8)	186 (7.3)	0.521
Thoracic vertebra	69 (18.2)	164 (15.4)	0.202	212 (14.2)	214 (14.4)	0.881	281 (15.0)	378 (14.8)	0.850
Lumbar vertebra	53 (14.0)	150 (14.1)	0.962	204 (13.7)	211 (14.2)	0.678	257 (13.8)	361 (14.2)	0.687
Sacral vertebra	24 (6.3)	78 (7.3)	0.518	125 (8.4)	131 (8.8)	0.670	149 (8.0)	209 (8.2)	0.785
Didymous bone									
Skull	10 (2.6)	37 (3.5)	0.431	26 (1.7)	26 (1.8)	0.988	36 (1.9)	63 (2.5)	0.226
Clavicle	7 (1.8)	16 (1.5)	0.645	43 (2.9)	33 (2.2)	0.253	50 (2.7)	49 (1.9)	0.095
Scapula	19 (5.0)	53 (5.0)	0.978	102 (6.8)	92 (6.2)	0.476	121 (6.5)	145 (5.7)	0.278
Humerus	11 (2.9)	44 (4.1)	0.283	60 (4.0)	66 (4.4)	0.569	71 (3.8)	110 (4.3)	0.392
Rib	51 (13.5)	153 (14.4)	0.662	185 (12.4)	195 (13.1)	0.554	236 (12.6)	348 (13.7)	0.320
Pelvic bone	58 (15.3)	155 (14.6)	0.724	198 (13.3)	200 (13.5)	0.880	256 (13.7)	355 (13.9)	0.827
Femur	19 (5.0)	92 (8.6)	0.023	114 (7.7)	119 (8.0)	0.709	133 (7.1)	211 (8.3)	0.155

Abbreviations: MBM, metachronous bone metastasis; SBM, synchronous bone metastasis.

^a^ Calculated by the chi‐squared test.

**TABLE 2:** Bone metastasis accumulated regions in synchronous or metachronous bone metastasis patients with nasopharyngeal carcinoma.

Bone metastasis site	Cohort 1			Cohort 2			Combined		
	SBM	MBM	*p*‐value^a^	SBM	MBM	*p*‐value^a^	SBM	MBM	*p*‐value^a^
	*N* = 112 (%)	*N* = 294 (%)		*N* = 328 (%)	*N* = 307 (%)		*N* = 440 (%)	*N* = 601 (%)	
First discovery									
1 region	44 (39.3)	109 (37.1)	0.681	104 (31.7)	101 (32.9)	0.748	148 (33.6)	210 (34.9)	0.661
2–3 region	33 (29.5)	84 (28.6)	0.859	99 (30.2)	102 (33.2)	0.410	132 (30.0)	186 (30.9)	0.743
4–5 region	20 (17.9)	46 (15.6)	0.589	46 (14.0)	53 (17.3)	0.261	66 (15.0)	99 (16.5)	0.520
6 region	15 (13.4)	55 (18.7)	0.205	79 (24.1)	51 (16.6)	0.020	94 (21.4)	106 (17.6)	0.132
Subsequent discovery									
0 region	84 (75.0)	248 (84.3)	0.029	221 (67.4)	141 (45.9)	<0.001	305 (69.3)	389 (64.7)	0.120
1 region	12 (10.7)	18 (6.1)	0.114	38 (11.6)	59 (19.2)	0.008	50 (11.4)	77 (12.8)	0.481
2–3 region	12 (10.7)	14 (4.8)	0.029	40 (12.2)	56 (18.2)	0.034	52 (11.8)	70 (11.6)	0.933
4 region	4 (3.6)	14 (4.8)	0.602	29 (8.8)	51 (16.6)	0.003	33 (7.5)	65 (10.8)	0.070
Total discovery									
1 region	34 (30.4)	92 (31.3)	0.856	61 (18.6)	59 (19.2)	0.842	95 (21.6)	151 (25.1)	0.185
2–3 region	31 (27.7)	85 (28.9)	0.806	99 (30.2)	63 (20.5)	0.005	130 (29.5)	148 (24.6)	0.076
4–5 region	25 (22.3)	45 (15.3)	0.094	49 (14.9)	63 (20.5)	0.065	74 (16.8)	108 (18.0)	0.629
6 region	22 (19.6)	72 (24.5)	0.301	119 (36.3)	122 (39.7)	0.369	141 (32)	194 (32.3)	0.936

Abbreviations: MBM, metachronous bone metastasis; SBM, synchronous bone metastasis.

^a^ Calculated using the chi‐squared test.

### 3.3 Survival outcomes

**FIGURE 3:**
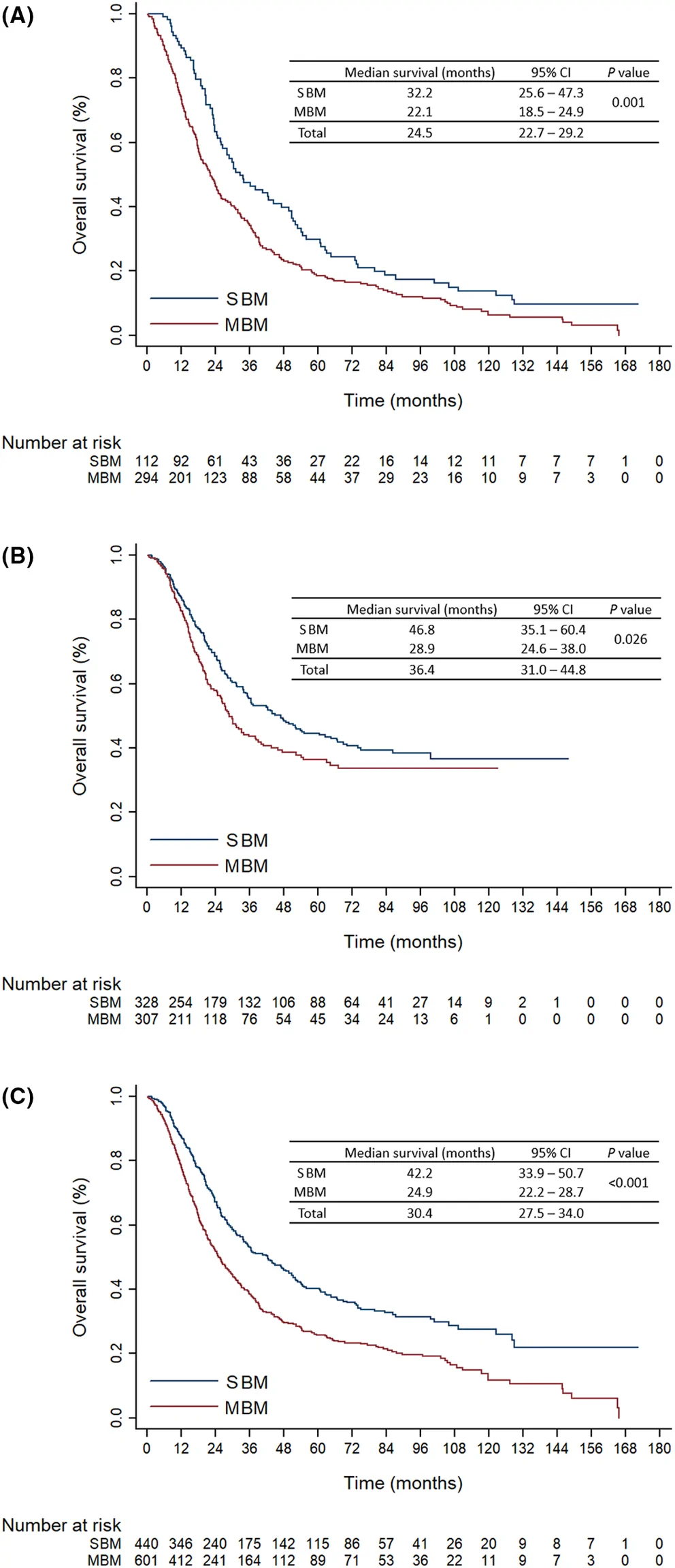
Kaplan–Meier overall survival curves in patients with nasopharyngeal carcinoma grouped based on the presence of synchronous versus metachronous bone metastasis. (A) Cohort 1; (B) Cohort 2; (C) Combined cohort 1 and cohort 2. MBM, metachronous bone metastasis; SBM, synchronous bone metastasis. Median survival was calculated using the Kaplan–Meier method. The *p*‐value was calculated using log‐rank test. 95% CI, 95% confidence interval.

We found that cohort 2 had significantly better OS (36.4 months, 95% CI: 31.0–44.8) compared with cohort 1 (24.5 months, 95% CI, 22.7–29.2) (Figure 3A,B). The SBM group (32.2 months) had significantly better OS than the MBM group (22.1 months; *p* = 0.001; Figure 3A). The SBM group also had better 1‐year (89.3% vs. 73.4%), 3‐year (47.5% vs. 34.1%), and 5‐year (29.8% vs. 18.5%) OS compared with the MBM group. In cohort 2, the SBM group (46.8 months) also had significantly better OS compared with the MBM group (28.9 months*; p* = 0.026; Figure 3B). The SBM group also had better 1‐year (86.6% vs. 82.4%), 3‐year (55.3% vs. 43.6%), and 5‐year (44.5% vs. 36.3%) OS compared with those in MBM patients. In a combined analysis of cohort 1 and cohort 2, the SBM group (42.2 months) also had significantly better OS compared with the MBM group (24.9 months; *p* < 0.001; Figure 3C). The SBM group also had better 1‐year (87.3% vs. 77.9%), 3‐year (53.1% vs. 38.5%), and 5‐year OS (40.2% vs. 25.8%) compared with the MBM group. Univariate analysis showed that older age at the time of the discovery of bone metastasis, metastasis to other organs, and a greater number of affected regions for bone metastasis were all significantly correlated with a poorer prognosis in both cohorts. In a multivariate analysis, these factors remained independently associated with an unfavorable OS across both cohorts. In the combined analysis, the prognostic factors associated with higher mortality risk were consistent with those identified above (Table 3). The sex and histological subtype did not affect the survival whether in SBM or in MBM groups (Table S3 and S4).

**TABLE 3:** Cox proportional HRs and 95% CIs for the association of prognostic factors with mortality in bone metastasis patients with nasopharyngeal carcinoma.

Prognostic factors	Cohort 1		Cohort 2		Combined	
	HRs (95% CIs)	*p*‐value^a^	HRs (95% CIs)	*p*‐value^a^	HRs (95% CIs)	*p*‐value^a^
Univariate						
SBM versus PBM	1.51 (1.18–1.93)	0.001	1.29 (1.03–1.62)	0.026	1.56 (1.33–1.83)	<0.001
Sex (female vs. male)	0.91 (0.66–1.24)	0.539	1.16 (0.87–1.53)	0.312	1.01 (0.82–1.25)	0.110
Age (per year)^b^	1.01 (1.00–1.02)	0.005	1.01 (1.00–1.02)	0.003	1.02 (1.00–1.02)	<0.001
With other organ metastasis (yes vs. no)	1.44 (1.15–1.80)	0.001	2.18 (1.70–2.78)	<0.001	1.80 (1.53–2.12)	<0.001
No. of bone metastasis region (per one)^c^	1.11 (1.06–1.16)	<0.001	1.14(1.09–1.18)	<0.001	1.12 (1.09–1.15)	<0.001
Multivariate						
SBM versus PBM	1.45 (1.12–1.86)	0.004	1.22 (0.97–1.55)	0.091	1.51 (1.28–1.78)	<0.001
Sex (female vs. male)	0.91 (0.67–1.25)	0.570	1.07 (0.81–1.42)	0.633	0.99 (0.80–1.22)	0.916
Age (per year)^b^	1.02 (1.01–1.03)	0.001	1.01 (1.00–1.03)	0.004	1.02 (1.01–1.02)	<0.001
With other organ metastasis (yes vs. no)	1.31 (1.04–1.65)	0.020	1.96 (1.52–2.52)	<0.001	1.59 (1.34–1.88)	<0.001
No. of bone metastasis region (per one)^c^	1.10 (1.05–1.15)	<0.001	1.13 (1.08–1.17)	<0.001	1.11 (1.08–1.15)	<0.001

Abbreviations: CIs, confidence intervals; HR, hazard ratios; MBM, metachronous bone metastasis; SBM, synchronous bone metastasis.

^a^ Calculated using the multivariate Cox regression.

^b^ The age at discovery of bone metastasis.

^c^ The number of bone metastasis regions at the time of first discovery.

### 3.4 Distribution of bone metastasis time

**FIGURE 4:**
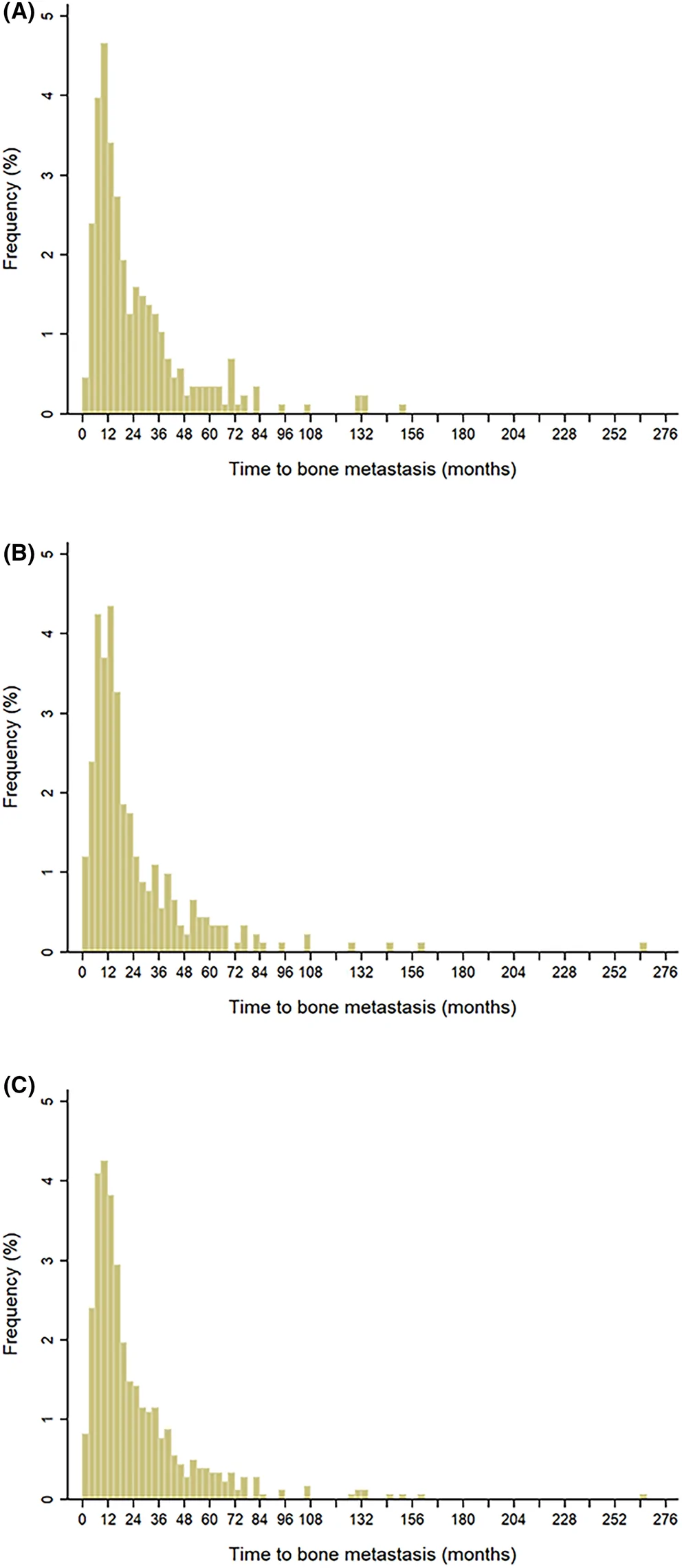
Frequency of bone metastasis occurring after the diagnoses of NPC. (A) Cohort 1; (B) Cohort 2; (C) Combined cohort 1 and cohort 2. The distributions of frequency of bone metastasis had no difference between cohort 1 and cohort 2. *p*‐value calculated by the Wilcoxon rank sum test between cohort 1 and cohort 2 was 0.339. The wide of bar is 3 months.

Most MBM occurred in between 7 and 18 months after diagnosing with NPC (Figure 4). The accumulated occurrence rates of MBM were 35%, 66%, and 80% at the end of 1, 2, and 3 years after diagnoses, respectively. The variable of time to metastasis in this study presented a positive skewness distribution. There was no difference in the distributions of frequency of bone metastasis between cohort 1 and cohort 2 (*p* = 0.339), which compared by Wilcoxon rank sum test. Besides that, we transformed these variables to normal distribution by tiwce Box‐Cox changes. The transformed variables were performed normality test by skewness test, kurtosis test, and equal variances test. The *p*‐values were 1, 0.095, and 0.076, respectively. And finally, there was no difference in the time to bone metastatic distribution between cohorts 1 and 2 according to the *t*‐test (*p* = 0.179) (Figure S2).

## 4 DISCUSSION

The NPC patients with distant metastases generally have an unfavorable prognosis and poor OS.^14^ The bone is the most frequently involved organ in patients with NPC who developed distant metastases.^7^, ^8^ Thus, it is critical to learn more about bone metastases in affected patients and expose the time and location of NPC bone metastasis, which will serve as a foundation for our follow‐up examinations and early identification of metastasis, as well as guiding the selection of appropriate treatment. To the best of our knowledge, this is the first prospective study to compare the distribution of bone metastases between patients with SBM versus MBM.

NPC is more common in males, with a sex ratio of approximately 3:1.^1^, ^3^, ^4^ Consistent with previous studies, 75% of patients in our cohort were male. However, male predominance was more obvious in patients with distant metastasis, especially in those with synchronous metastasis. In the SBM group, >85% of patients were male. Males are more likely to develop distant metastasis, which may explain why male patients with NPC also tend to have a poor prognosis.^1^, ^3^, ^4^ Previous study has proposed when initially diagnosed with NPC, bone scans should perform in all male and N2‐3 female patients with high EBV DNA to screen bone metastasis.^15^

Few studies have explored the characteristics of bone metastases in patients with NPC. A retrospective study that enrolled patients with bone‐only metastatic NPC found that the rib, thoracic vertebrae, and lumbar vertebrae were the most sites of involvement.^12^ Our study showed that the thoracic vertebrae, lumbar vertebrae, pelvic bones, and ribs were the most common sites of bone metastasis. These areas all have a rich blood supply and many cancellous bones, which are suitable for the growth of metastatic tumors.

Our study further compared the distribution of bone metastases between patients with SBM and MBM. Cervical vertebra metastases were more frequent in the SBM group than the MBM group. This relationship may be due to the cervical vertebra being located near the nasopharynx. Interestingly, cervical metastases were less common in the follow‐up. It may be due to dispersed radiation in nasopharyngeal and neck irradiation for nasopharyngeal carcinoma; the cervical spine can be exposed to doses of 40 to 50 Gy, suppressing subclinical lesions and decreasing the risk of cervical metastases during follow‐up. A retrospective study found no statistically significant differences in OS between patients with involvement of pterygoid structures alone and those with erosion of the skull base and/or cervical vertebra.^16^ Based on this result, cervical vertebral invasion was added as T3 in the 8th edition of the TNM staging system.^17^ Cervical vertebral invasion is no longer considered a distant metastasis but part of a primary tumor. In this study, patients who were older at the discovery of bone metastasis, the presence of other visceral metastases, and a greater number of involved site were associated with worse OS. These findings are similar with those of other studies.^18^, ^19^, ^20^ It is not hard to understand that multiple lesions reflect a heavier tumor burden and more active tumor cells, which bring about a poor prognosis for patients.

The aim of our study was to compare the prognosis of patients with bone metastasis at initial diagnosis with those of patients who developed bone metastasis after radical chemoradiotherapy. We discovered that the MBM group had a worse OS compared with the SBM group, indicating that patients with synchronous bone metastases are deserving of using the initiative treatments. We speculate that the reasons for this finding are as follows. First, more patients in the MBM group than the SBM group had metastasis to other organs, which was correlated with poor OS. Second, the tumor cell clones in the MBM group could survive after radical chemoradiotherapy, implying that they may be insensitive or even resistant to chemoradiotherapy, which may lead to poor subsequent treatment results. By contrast, the median OS of patients improved from 32.2 months before 2010 to 46.8 months after 2011. Recent randomized phase 3 trials remind us that chemotherapy combined locoregional radiotherapy with significantly improves OS in chemotherapy‐sensitive patients with metastatic NPC.^21^ Capecitabine maintenance therapy also significantly improves PFS in patients with newly diagnosed metastatic NPC.^22^ The addition of immunotherapy (toripalimab) to GP chemotherapy for NPC patients with metastasis provided superior PFS compared with GP alone.^23^ Therefore, patients with bone metastasis at diagnosed NPC still have opportunity to treat actively. The study showed that these patients received more than 6 cycles of chemotherapy would have better survival and prognosis.^24^ But after when these patients had been treated by palliative chemotherapy plus locoregional radiotherapy, further local radiotherapy to bone metastases would not significantly improve survival.^25^, ^26^, ^27^ If performed radiotherapy to metastatic bones, the radiation dose should give radical dose rather than palliative dose.^28^ Zoledronic acid would prevent bone‐related events, but could not improve OS.^29^

Our findings, which show a tendency for most metachronous bone metastasis to occur in between 7 and 18 months after being diagnosed with NPC, are in line with those of a study, which found that the incidence of bone metastasis in patients with solid cancer increased mostly in the first 2 years.^30^ As a result, during follow‐up, paying more attention to check for bone metastases by bone ECT, PET/CT, MR, or CT may enable early detection and treatment of bone metastases, increasing patient survival as a whole. One study have recommended imaging at follow‐up should be considered between 7 and 36 months.^31^ This was consistent with us data which showed that the accumulated occurrence rate of MBM was 80% in the first 3 years. Another study reminded us to pay attention to the patients with skull base invasion, because these patients were at risk of bone metastasis.^32^ For patients with locoregionally advanced NPC, after receiving standard‐of‐care treatment, metronomic capecitabine chemotherapy sustaining 1 year significantly improved failure‐free survival.^33^ In our data, the accumulated occurrence rate of MBM was 35% in the first 1 year, so 1‐year metronomic capecitabine chemotherapy should be needed.

In this study, several patients were diagnosed and treated in the early part of this century, and only a small number of patients had data on plasma EBV DNA load at diagnosis of metastasis; therefore, this biomarker was not included in our analysis. In addition, the treatments applied in patients with NPC and metastases were varied; therefore, we did not include a therapeutic regimen to analyze their effects on prognosis.

## 5 CONCLUSIONS

This is the first large‐scale cohort study to compare the distribution characteristics of bone metastasis between patients with SBM compared with MBM after radical chemoradiotherapy. Radical radiotherapy interventions did not change the NPC bone metastasis path or destination. SBM tended to metastasize to the cervical vertebra, possibly due to the bone being located close to the nasopharynx. Our findings provide more evidence for clinical decision‐making in patients with NPC bone metastasis, which warrants more initiative treatment following initial diagnostic confirmation. Furthermore, we found a tendency for most metachronous bone metastasis to occur in between 7 and 18 months after being diagnosed with NPC and suggest that take care to check for bone metastasis during this time.

## AUTHOR CONTRIBUTIONS

**Dong‐Hua Luo:** Conceptualization (lead); writing – original draft (lead). **Jia‐Xin Li:** Writing – original draft (equal). **Wan‐Ping Guo:** Data curation (equal). **Chen‐Guang Guo:** Data curation (equal). **Xiao‐Han Meng:** Data curation (equal). **Pei‐Jun Xie:** Data curation (equal). **Jie‐Yi Lin:** Data curation (equal). **Hao‐Yuan Mo:** Supervision (equal). **Qun Zhang:** Data curation (equal). **yong chen:** Data curation (equal); validation (equal); writing – review and editing (equal). **Guo‐Ping Shen:** Conceptualization (equal); writing – original draft (equal).

## FUNDING INFORMATION

This work was supported by Guangzhou Science and Technology Program Project (No. 202201010823).

## CONFLICT OF INTEREST STATEMENT

None.

## ETHICS APPROVAL STATEMENT AND PATIENT CONSENT STATEMENT

This study was approved by the Human Ethics Approval Committee of Sun Yat‐sen University Cancer Center (No. YB2005001), and informed consents were obtained from all patients.

## STATEMENTS RELATING TO ETHICS AND INTEGRITY POLICIES

Neither the entire paper nor any parts of its content has been published or has been under consideration elsewhere. It is not being submitted to any other journal. We declare no conflicts of interest (either financial or personal) and the content of the manuscript is original. All authors have contributed to, read, and approved the final manuscript for submission.

## Supporting information


Figure S1–S2.

Table S1–S4.

## Data Availability

Due to the nature of this research, participants of this study did not agree for their data to be shared publicly, so supporting data are not available.
