# Supplementary material for: The map of bone metastasis in nasopharyngeal carcinoma: A real‐world study
Source: Cancer Med. 2023 Aug 10;12(17):17660–70. doi: 10.1002/cam4.6383 (PMC10523956; doi:10.1002/cam4.6383)
Supplement: Supplementary file 1 — Figure S1–S2. [file CAM4-12-17660-s001.docx]

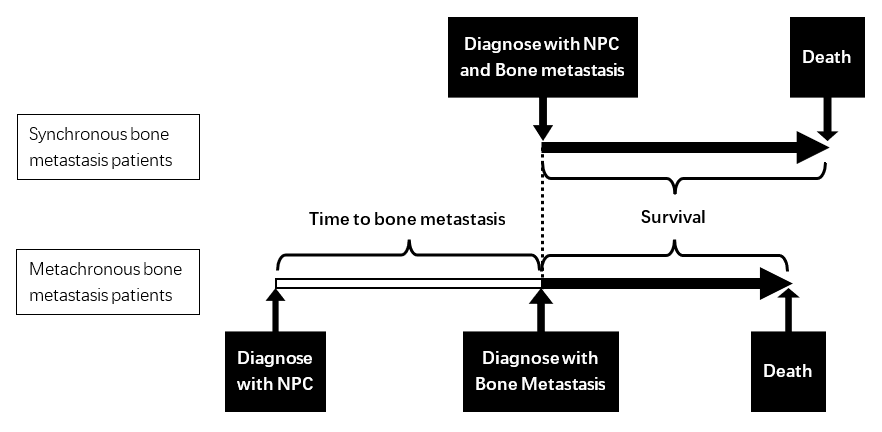


eFigure 1. The overall survival (OS) was defined as the interval between the date of diagnosis of bone metastases and the date of the last visit or death. The time to metastasis was defined as the interval between the date of diagnosis of NPC and the date of discovery of bone metastases.


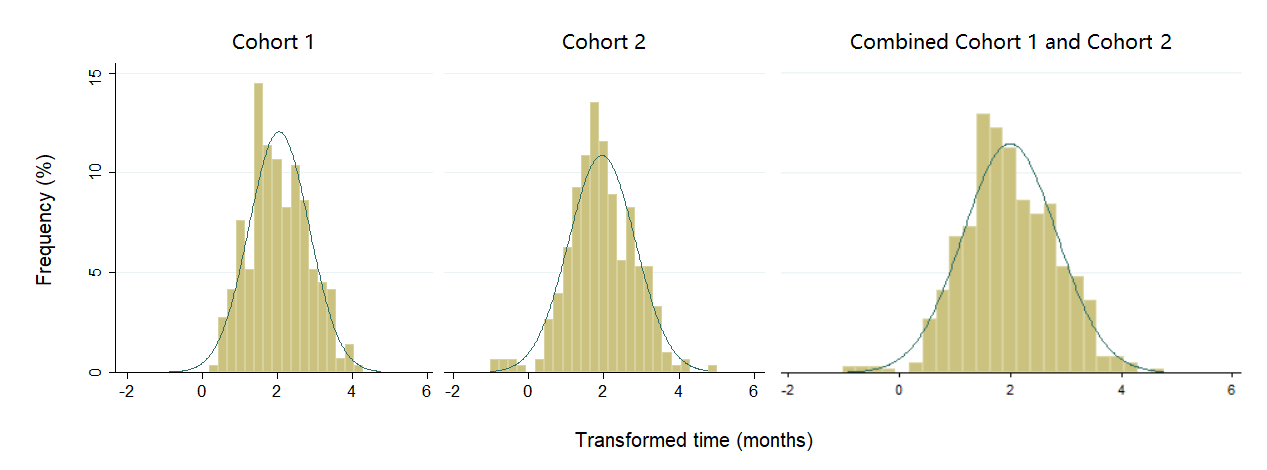


eFigure 2. The variable of time to bone metastasis presented positive skewness distribution. These variables were transformed to normal distribution by twice Box-Cox change. The P-values of transformed variables in skewness test, kurtosis test, and equal variances test were 1, 0.095, and 0.076, respectively. The P-value of t-test between cohort 1 and cohort 2 was 0.179. It means the time to bone metastasis distribution between cohort 1 and cohort 2 have no difference.
