# Supplementary material for: The map of bone metastasis in nasopharyngeal carcinoma: A real‐world study
Source: Cancer Med. 2023 Aug 10;12(17):17660–70. doi: 10.1002/cam4.6383 (PMC10523956; doi:10.1002/cam4.6383)
Supplement: Supplementary file 2 — Table S1–S4. [file CAM4-12-17660-s002.docx]

| eTable 1. Characteristics of nasopharyngeal carcinoma cohort. | | | | | | | | | |
| --- | --- | --- | --- | --- | --- | --- | --- | --- | --- |
| Characteristics | Cohort1  N=4366 (%) | SBM  N=112 (%) | MBM  N=294 (%) | P value^a^ | Cohort2  N= 5976(%) | | SBM  N=328 (%) | MBM  N=307 (%) | P value^a^ |
| Sex |  |  |  |  |  |  | |  |  |
| Male | 3295 (75.5) | 103 (92.0) | 245 (83.3) | 0.026 | 4414 (73.9) | 279 (85.1) | | 250 (81.4) | 0.221 |
| Female | 1071 (24.5) | 9 (8.0) | 49 (16.7) |  | 1562 (26.1) | 49 (14.9) | | 57 (18.6) |  |
| Age (years) |  |  |  |  |  |  | |  |  |
| ＜46 | 2237 (51.2) | 54 (48.2) | 123 (41.8)^b^ | 0.247 | 3324 (55.6) | 160 (47.8) | | 159 (51.8)^b^ | 0.448 |
| ≥46 | 2129 (48.8) | 58 (51.8) | 171 (58.2)^b^ |  | 2652 (44.4) | 168 (51.22) | | 148 (48.2)^b^ |  |
| Clinical stage |  |  |  |  |  |  | |  |  |
| Ⅰ | 230 (5.3) | 0 | 3 (1.0) | NA | 280 (4.7) | 0 | | 1 (0.3) | NA |
| Ⅱ | 880 (20.2) | 0 | 31 (10.5) |  | 1262 (21.1) | 0 | | 28 (9.1) |  |
| Ⅲ | 1893 (43.4) | 0 | 144 (49.0) |  | 2764 (46.3) | 0 | | 143 (46.6) |  |
| Ⅳa | 1205 (27.6) | 0 | 116 (39.5) |  | 1419 (23.7) | 0 | | 135 (44.0) |  |
| Ⅳb | 158 (3.6) | 112 (100.0) | 0 (0.0) |  | 251 (4.2) | 328 (100.0) | | 0 (0.0) |  |
| Initial treatment |  |  |  |  |  |  | |  |  |
| RT alone | 1193 (27.3) | 3 (2.7) | 48 (16.3) | NA | 1596 (26.7) | 0 (0.0) | | 0 (0.0) | NA |
| RT+CT | 1114 (25.5) | 38 (33.9) | 108 (36.7) |  | 1410 (23.6) | 85 (25.9) | | 108 (35.2) |  |
| CRT | 1868 (42.8) | 36 (32.1) | 127 (43.2) |  | 2731 (45.7) | 108 (32.9) | | 193 (62.9) |  |
| CT | 191 (4.37) | 35 (31.3) | 11 (3.7) |  | 239 (4.0) | 135 (41.2) | | 6 (1.9) |  |
| Metastasis |  |  |  |  |  |  | |  |  |
| No | 3564 (81.6) | 0 | 0 |  | 4725 (79.1) | 0 | | 0 |  |
| Bone | 406 (9.3) | 112 | 294 |  | 635 (10.63) | 328 | | 307 |  |
| Only bone | 174 (4.0) | 60 (53.6) | 114 (38.8) | 0.007 | 255 (4.27) | 159 (48.5) | | 96 (31.3) | <0.001 |
| With other organs | 232 (5.3) | 52 (46.4) | 180 (61.2) |  | 380 (6.36) | 169 (51.5) | | 211 (68.7) |  |
| Other organs | 396 (9.1) | 0 | 0 |  | 616 (10.3) | 0 | | 0 |  |
| Follow-up time (months) |  |  |  |  |  |  | |  |  |
| Median | – | 27.0 | 18.7 | NA | – | 27.4 | | 18.1 | NA |
| Range | – | 0.1 – 172.4 | 0.05 – 165.7 |  | – | 1.5 – 147.8 | | 0.1 – 123.1 |  |
| Note: SBM, synchronous bone metastasis. MBM, metachronous bone metastasis. NA, not applicable. RT, radiotherapy. CT, chemotherapy. CCRT, concurrent chemoradiotherapy. a. Calculated by the chi-square test. b. The age at discovery of bone metastasis. | | | | | | | | | |

| eTable 2. Initial method of radiotherapy in nasopharyngeal carcinoma cohort. | | | | | | | | |
| --- | --- | --- | --- | --- | --- | --- | --- | --- |
| Characteristics | Cohort 1  N=360 (%) | SBM  N=77 (%) | MBM  N=283 (%) | P value | Cohort 2  N= 494(%) | SBM  N=193 (%) | MBM  N=301 (%) | P value |
| Method of radiotherapy |  |  |  |  |  |  |  |  |
| 2D-CRT | 338 (93.9) | 75 (97.4) | 263 (92.9) | 0.1466 | 142 (28.7) | 24 (12.4) | 118 (39.2) | <0.001 |
| IMRT | 22 (6.1) | 2 (2.6) | 20 (7.1) |  | 352 (71.3) | 169 (87.6) | 183 (60.8) |  |
| Note: SBM, synchronous bone metastasis. MBM, metachronous bone metastasis. 2D-CRT, two-dimensional conventional radiotherapy. IMRT, intensity-modulated radiation therapy. P values were calculated by the chi-square test. | | | | | | | | |

| eTable 3. Median survival in nasopharyngeal carcinoma cohorts between male and female. | | | | | | |
| --- | --- | --- | --- | --- | --- | --- |
|  | Cohort 1 | | Cohort 2 | | Combined | |
|  | SBM  N=112 | MBM  N=294 | SBM  N=328 | MBM  N=307 | SBM  N=440 | MBM  N=601 |
| Sex |  |  |  |  |  |  |
| Male | 103 (92.0) | 245 (83.3) | 279 (85.1) | 250 (81.4) | 382 (86.8) | 495 (82.4) |
| Female | 9 (8.0) | 49 (16.7) | 49 (14.9) | 57 (18.6) | 58 (13.2) | 106 (17.6) |
| Median survival |  |  |  |  |  |  |
| Male | 33.7 (26.5-47.3) | 22.1 (18.4-25.6) | 44.8 (35.1-60.4) | 31.0 (24.6-40.7) | 42.2 (33.7-49.8) | 24.9 (22.2-29.8) |
| Female | 23.7 (8.3-NA) | 24.3 (16.5-39.2) | 49.8 (28.0-NA) | 26.3 (16.9-30.1) | 49.8 (25.5-NA) | 24.5 (18.3-31.8) |
| P-value | 0.302 | 0.654 | 0.951 | 0.185 | 0.396 | 0.606 |
| Note: SBM, synchronous bone metastasis. MBM, metachronous bone metastasis. Median survival was calculated using the Kaplan–Meier method and its unit was month. The p-value was calculated using log-rank test. Parentheses were filled median survival 95% confidence interval. NA, not available. | | | | | | |

| eTable 4. Histological subtypes and median survival in nasopharyngeal carcinoma cohorts. | | | | | | |
| --- | --- | --- | --- | --- | --- | --- |
|  | Cohort 1 | | Cohort 2 | | Combined | |
|  | SBM  N=112 | MBM  N=294 | SBM  N=328 | MBM  N=307 | SBM  N=440 | MBM  N=601 |
| WHO classification |  |  |  |  |  |  |
| NKUC | 102 (91.1) | 273 (92.9) | 322 (98.2) | 304 (99.0) | 424 (96.4) | 577 (96.0) |
| NKDC | 9 (8.0) | 21 (7.1) | 2 (0.6) | 1 (0.3) | 11 (2.5) | 22 (3.7) |
| KSCC | 1 (0.9) | 0 (0) | 4 (1.2) | 2 (0.7) | 5 (1.1) | 2 (0.3) |
| Median survival |  |  |  |  |  |  |
| NKUC | 33.9 (26.0-49.8) | 22.2 (18.4-25.6) | 44.8 (35.1-60.4) | 28.7 (24.6-35.6) | 42.2 (34.0-50.7) | 24.9 (22.1-28.7) |
| NKDC+KSCC | 25.6 (7.1-NA) | 22.0 (12.3-51.3) | NA (8.8-NA) | NA (NA-NA) | 26.5 (9.1-NA) | 24.5 (12.5-52.2) |
| P-value | 0.774 | 0.850 | 0.290 | 0.132 | 0.784 | 0.693 |
| Note: SBM, synchronous bone metastasis. MBM, metachronous bone metastasis. Median survival was calculated using the Kaplan–Meier method and its unit was month. The p-value was calculated using log-rank test. Parentheses were filled median survival 95% confidence interval. WHO, World Health Organization. NKUC, non-keratinizing undifferentiated carcinoma. NKDC, non-keratinizing differentiated carcinoma. KSCC, keratinizing squamous cell carcinoma. NA, not available. | | | | | | |
